# 3E enhancement of freshwater productivity of solar still with heater, vibration, and cover cooling

**DOI:** 10.1007/s11356-022-20340-9

**Published:** 2022-04-30

**Authors:** Mohamed M. Khairat Dawood, Abubakr Helmy Omar, Ali Ismail Shehata, Ahmed Samir Shehata, Ahmed Abd-Elsalam Taha, Mohamed Nabil El-Shaib, Madeha Kamel Mohamed

**Affiliations:** 1 Mechanical Engineering Department, Faculty of Engineering, Suez Canal University, Ismailia, Egypt; 2 Mechanical Engineering Department, Arab Academy for Science, Technology and Maritime Transport, Alexandria, Egypt; 3 Marine Engineering Department, Arab Academy for Science, Technology and Maritime Transport, Alexandria, Egypt

**Keywords:** Freshwater, Solar still, Thermoelectric cooler, Vibration, Daily productivity, Solar radiation, Enhancement

## Abstract

This study focused on experimentally increasing the productivity of freshwater from solar stills. The performance of a single solar still system could be augmented with the combination of an electric heater, vibration motion, and thermoelectric cooling. The study investigated the effects of combining two of these components and finally combining all of them on freshwater productivity. The electric heater and vibration motion are used to enrich the evaporation rate, while thermoelectric coolers are used to enhance the condensation rate, leading to high freshwater productivity. The proposal, construction, and testing of two identical solar stills were performed under the local climate conditions of the city of Alexandria in northwestern Egypt during the summer and winter times. The two solar stills had a 1-m^2^ base area. An electric heater of 450 W was placed inside the modified solar still. The modified solar still was fixed on four coiled springs. A 1-hp power DC motor, an inverter, a control unit, and two 330-W photovoltaic solar panels were attached to the modified solar still. Eccentric masses were mounted on the rotating disk attached to the DC motor to generate the vibration. Under the same climate conditions, the daily output of freshwater was measured experimentally for the modified case and the conventional solar. The daily rates of freshwater productivity in summer were investigated for four cases and the conventional one. Results showed that the peak daily freshwater productivity achieved with the solar heater, thermoelectric coolers, and vibration motion was 12.82 kg/day, with a maximum estimated cost of 0.01786 $/L/m^2^.The exergoeconomic of the modified solar still with heater, vibration, and thermoelectric cooler was greater than that of conventional ones. The highest CO_2_ mitigation of the case (5) and that of the conventional solar desalination were about 160 tons and 28 tons, respectively.

## Introduction

The shortage of clean water for daily use in many parts of the world is an important problem and requires urgent attention (Selvaraj and Natarajan 2018). The situation is especially difficult in third world countries because of the population explosion and industrial and agricultural development (Boubekri et al. 2013; Sampathkumar et al. 2010). Enhancing the effectiveness and efficiency of water purification technology is widely considered the major challenge of the twenty-first century (Elimelech 2006), especially in Egypt where the constructed El-Nahda dam in Ethiopia is expected to affect the percentage of freshwater available. Therefore, intensive efforts are underway throughout the world to avoid this crisis; these efforts include the conservation of the existing limited freshwater supply and the conversion of the amply available seawater through several desalting technologies (Sharshir et al. 2016). The problem of freshwater supply can be resolved by the desalination of seawater (Eltawil et al. 2009). The decreased freshwater is not the only reason behind the forced application of water desalination technology. The severe pollution of lakes, rivers, and groundwater, in some cases, has significantly reduced the quality of available freshwater sources (El-Bialy et al. 2016). Places with an ample supply of solar radiation and brackish water, such as Egypt, are able to produce a sensible amount of drinkable water at economical costs (El-Bialy et al. 2016) via solar stills that are easy to build and are relatively inexpensive (Muftah et al. 2014). A solar still is an unpretentious device used to purify water using solar energy through the simple principle of evaporation followed by condensation (Arunkumar et al. 2019; Suneesh et al. 2014). Solar stills are classified into two separate types, namely, active and passive (SINGH et al. 1996). For passive stills, the water in a basin is subjected to direct heating, thereby eliminating the need for external heating sources. With these devices, distillation and heat collection occur within a single system. For active solar stills, the water undergoes direct heating as well, but these devices also use preheated water resulting from an indirect process involving external heating (Abdullah et al. 2021) to increase the water temperature in the basin and thereby automatically increase the evaporation rates (Muftah et al. 2014). Passive solar stills are used for solar distillation plants worldwide because of their simple fabrication and operation and their low cost; however, their production is low. Nevertheless, several active techniques have been developed to address this deficiency (Sampathkumar and Senthilkumar, 2012). The production cost per liter of drinkable water using either passive or active solar desalination mechanics is more economical than the price per liter in Egyptian markets (El-Bialy et al. 2016). Desalination using solar stills is made simple by several techniques for salinity removal. Meanwhile, researchers have consistently aimed to improve the productivity of solar stills (Abujazar et al. 2016; Edalatpour et al. 2016; Selvaraj and Natarajan 2018). Several types of active and passive solar stills (Kalogirou 2005) have been developed (Kabeel and El-Agouz 2011; Malik 1982), but their productivity remains problematic; hence, studies have attempted to vary the parameters affecting the efficiency rates of these devices (Bait and Si–Ameur 2018; Boubekri et al. 2013; Pansal et al. 2020; Sharshir et al. 2019; Sivakumar and Ganapathy Sundaram 2013). For productivity enhancement, increasing the basin water temperature by using a water heater is the best option (Al-Garni 2012b; Ben Bacha et al. 2007; Budihardjo et al. 2007; Han et al. 2010; Iqbal et al. 2018; Voropoulos et al. 2004). Shahin Shoeibi and his team (Shoeibi et al. 2021c) want to explore how the use of thermoelectric cooling and heating with different nanofluids affects the performance of solar stills. They found that solar stills with thermoelectric cooling and heating and Al_2_O_3_, TiO_2_, CuO, and MWCNT nanofluids at a concentration of 0.9% boosted water productivity by 11.57, 7.16, 6.32, and 4.66%, respectively, when compared to solar stills without nanofluid. Another study used porous media, nano-enhanced phase transition material (Shoeibi et al. 2022a), and nano-enhanced absorption to improve the efficacy of solar desalination (nano-coated) (Shoeibi et al. 2021a). CuO and Al_2_O_3_ nano-enhanced PCM at a concentration of 0.3 wt percent, and CuO nano-coated, respectively, boosted the productivity of solar stills by 55.8% and 49.5%, according to the study. Furthermore, nano-coating enhanced the solar still’s water output rate by roughly 5.7%.

The impact of using a solar water heater to boost the productivity and performance of a solar still was investigated experimentally by Sampathkumar and Senthilkumar (2012) on various days and with different timings. The productivity of the still increased the yield by 77% relative to the passive solar still. Thermoelectric cooling, heating, and power generators are all being looked at to improve the performance of solar energy systems in various ways. The hot side of the thermoelectric module contributes to raise the temperature of the solar working fluid in thermoelectric heating applications, such as desalination, water, or air heater systems (Shoeibi et al. 2022c). In the thermoelectric cooling situation, the cold side of the module helps to reduce the temperature of things like PV panels, desalination units’ condensation areas, and so on (Shoeibi et al. 2022b). According to Shoeibi et al. (2021d), the solar still with water glass cooling and PV/T had the highest enhanced water productivity, which was nearly 6 times greater than the standard sun still. Furthermore, a solar still with external condenser, PCM, and wick material had the lowest cost per liter, which was at 0.011$/L. Omara et al. (2013) conducted an experiment by using a new hybrid system, which included an evacuated solar water heater, a wick still, and a solar still. The water productivity of the hybrid system increased by 114% relative to the conventional double layer square wick solar still. The daily average efficiency of the double layer square wick still was 71.5%. During experimentation, the distillate water productivity increased by 215% when hot brackish water was fed during nighttime. Praveen kumar et al. (2018) compared the conventional and hybrid photovoltaic/thermal active solar stills in the climate conditions of Virudhnangar, India. They incorporated nickel chromium wire as a heater and a solar photovoltaic panel as a power source in a hybrid photovoltaic/thermal active solar still. The overall thermal and electrical efficiency increased by 25% relative to the conventional solar still (CSS). In another study, saline water in a single basin solar still was preheated in a water preheating system provided in the solar still that consisted of an absorber plate and riser tubes (Rajaseenivasan et al. 2014). The result revealed that the freshwater production rate accelerated by 60% relative to the CSS. A glass basin solar still was fabricated with an integrated water preheater and water evaporator section (Rajaseenivasan et al. 2016). The preheater contained hollow rectangular fins for holding the energy storage materials. The maximum daily yield was 3.61 kg when charcoal was used in the fins. Yadav and Sudhakar (2015) reviewed various domestic designs of solar stills and concluded that for a hybrid system integrated with an evacuated solar water heater, the maximum yield is around 12.48 L/m^2^ per day, and the range of maximum thermal efficiency is 17.4–45%. A comparative study on a single basin solar still with and without solar water heater integration and cover cooling was conducted (Morad et al. 2015). The maximum daily freshwater yields using active and passive solar stills with a solar water heater were 10.06 and 7.8 kg/m^2^, respectively. Various techniques are used for water heating in solar stills, and one example is the use of an induction water heating system. Furthermore, studies have extensively focused on water heating (G Manuel 2009), and they have developed induction water heaters (Unver 2012). Thus, an induction water heater system seems to be a useful innovation. An induction heater has high electrical and fire safety. The heating element has no electrical connection with the inductor. The maximum temperature on the surface of the heater exceeds the temperature of the heat carrier by not more than 10–30 °C (for heaters working in hot water supply systems). In many cases, the transition to induction electric heating reduces operating costs by an average of 30%. Moreover, induction heaters are easy to install and maintain, are compact in size, have minimal space requirements (Liu et al. 2019; Sampathkumar and Senthilkumar, 2012). Another important parameter for productivity enhancement is glass cooling for solar still covers (Omara et al. 2017). The productivity of a solar still is influenced by the temperature difference between the condensing and evaporating areas. Previous research has determined that increasing the difference between basin water and glass temperatures enhances the daily productivity of solar stills (Kalidasa Murugavel et al. 2008), which improves the vaporization rate, thereby leading to a high distilled water yield for solar stills. Therefore, glass temperature can be decreased using a cooling process (Rubioa et al. 2000; Setoodeh et al. 2011; TOYAMA et al. 1983). Kaushal and Varun (2010) highlighted that solar still efficiency increases by 20% when a 1.3-mm-thick cooling film is introduced to a 5-mm-thick glass cover. Meanwhile, Somwanshi and Tiwari (2014) studied experimentally the top cover cooling of a single basin solar still by using an air cooler for two different cooling methodologies in four various climatic zones in the Indian plains. The increase in annual production with water from the evaporative cooler was about 41.3–56.5%, and that with water at ambient temperature was about 30.1–21.8%. Furthermore, the influence of heat capacity on still efficiency with flowing water above the glass was studied by Lawrence et al. (1990). The results indicated increases in solar still efficiency from 7 to 10%. Sharshir et al. (2017a) and Sharshir et al. (2017b) studied experimentally the effects of using different cooling flow rates on the efficacy of solar stills and used some nanoparticles under glass cooling to increase still productivity. The economical flow rate of the cooling water above the glass was 4 kg/h. When the cooling water over the glass was used, the daily productivity improved by 57.60% and 47.80%, and the efficacy was enhanced remarkably by 49% and 46% with the graphite and copper oxide nanoparticles, respectively, relative to those obtained with the conventional basin. Srithar et al. (2016) experimentally studied the performance of a stand-alone triple basin solar system. The cover cooling enhanced the overall productivity by 32.8%. Abdullah (2013) performed experiments in a stepped still with top cover cooling. The flow rate of cooling water was 0.03 kg/s. By using hot air and glass cover cooling, the still efficiency increased by 65% and 53%, respectively. Integrating both techniques in the stepped solar still, the still efficiency improved by 112%. Moreover, using stepped solar desalination technology becomes an important solution for potable water shortage problems in hot arid areas such as Middle East, while such technology is inexpensive with low maintenance costs and less environmental impact compared with reverse osmosis technology (RO) (Shmroukh and Ookawara 2021). Every experimental and numerical investigation in the subject of solar water desalination includes an economic analysis. The environmental consequences and carbon credit earnings of these devices have been proven to be quite important in solar desalination research, in addition to the economic analysis. According to reference (Shoeibi et al. 2021b), the entire water production cost of solar stills was reported to be between 0.0014 and 0.29 $/L, with the maximum CO_2_ mitigation projected to be around 1129.53 tonnes in the solar still coupled with photovoltaic/thermal and solar collector during life duration. Furthermore, distilled water has superior quality characteristics than saline water for drinking, such as TDS, TSS, PH, EC, and turbidity (Shoeibi et al. 2022c). The use of the thermoelectric refrigeration concept (Rajput 2005) is expanding because of the low energy cost, easy installation, compact size, light weight, zero noise, and ease of use. Hence, scholars have employed thermoelectric cooler (TEC) modules to enhance the performance, efficacy, and productivity of solar desalination systems, such as solar stills (Esfahani et al. 2011; Nazari et al. 2019; Shoeibi et al. 2020). TECs can efficiently work with photovoltaic (PV) panels because of the low voltage requirement, and they can receive power supply directly from these panels. Thermoelectric devices are also insensitive to movement and are thus attractive for use in portable devices (Rajput 2005; Riffat and Ma 2003). The existing literature highlights the importance of investigating solar stills integrated with water heaters to increase the basin water temperature, in addition to the cooling techniques of solar stills’ glass covers for productivity enhancement. Therefore, the current work aims to investigate experimentally the productivity enhancement of solar stills under Egyptian climate conditions through thermoelectric glass cover cooling and heating and vibration motion to increase the evaporation rate. To the best of authors’ knowledge, this is the first time that the effect of integrated of three different techniques on the freshwater productivity has been investigated experimentally for the solar still. These techniques are (1) thermoelectric glass cover cooling, (2) water heating, and (3) vibration motion is used to increase the evaporation rate for high water temperature conditions, powered by solar PV to reduce the additional cost of extra thermal energy sources and under Egyptian climate conditions.

## Experimental setup

**Fig. 1:**
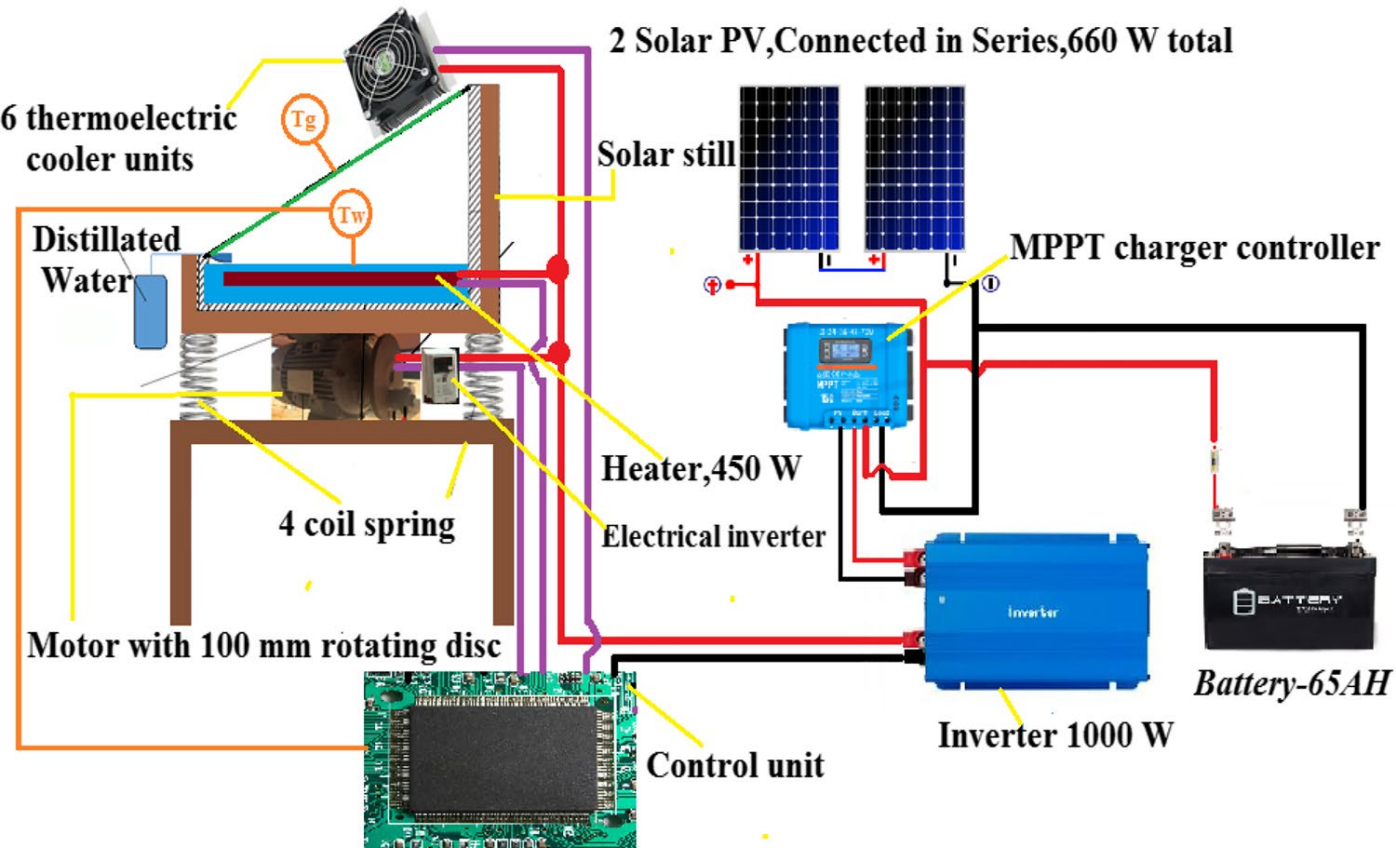
Schematic diagram for the proposed improved solar still

**Fig. 2:**
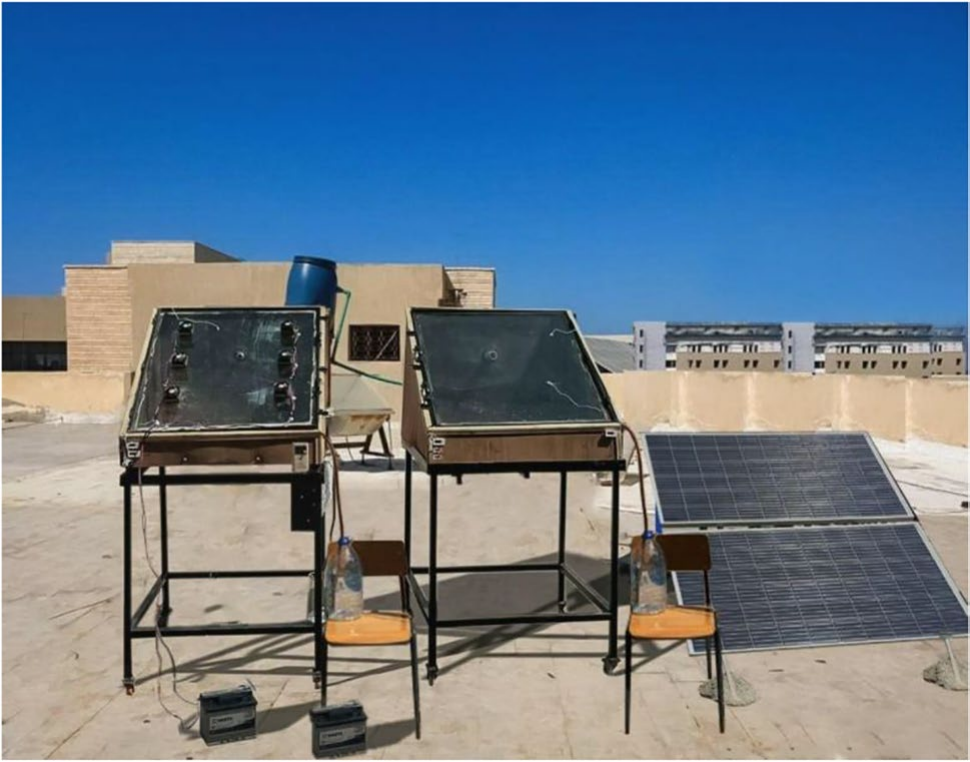
Photograph for the improved solar still and conventional solar still

Figure 1 presents the schematic of the improved solar still system, which comprises a single solar still, four coiled springs, a chassis, a DC motor, two polycrystalline PV solar panels (300 W), a battery (65 AH), an electric LS inverter M100 model, an electric heater (450 W), an MPPT charge controller, an inverter, a control, and six modules of TECs. Figure 2 shows an image of the test rig for the modified solar still and CSS with equal dimensions and specifications. The stills were made of 4-mm-thick galvanized steel with a square base measuring (1 × 1) m^2^. Thermal insulations were used to reduce the heat loss; 20-mm-thick polystyrene foam, 10-mm-thick glass wool, and a 5-mm-thick wooden layer were installed consecutively. The basin plate was painted black for good solar radiation absorption.

**Fig. 3:**
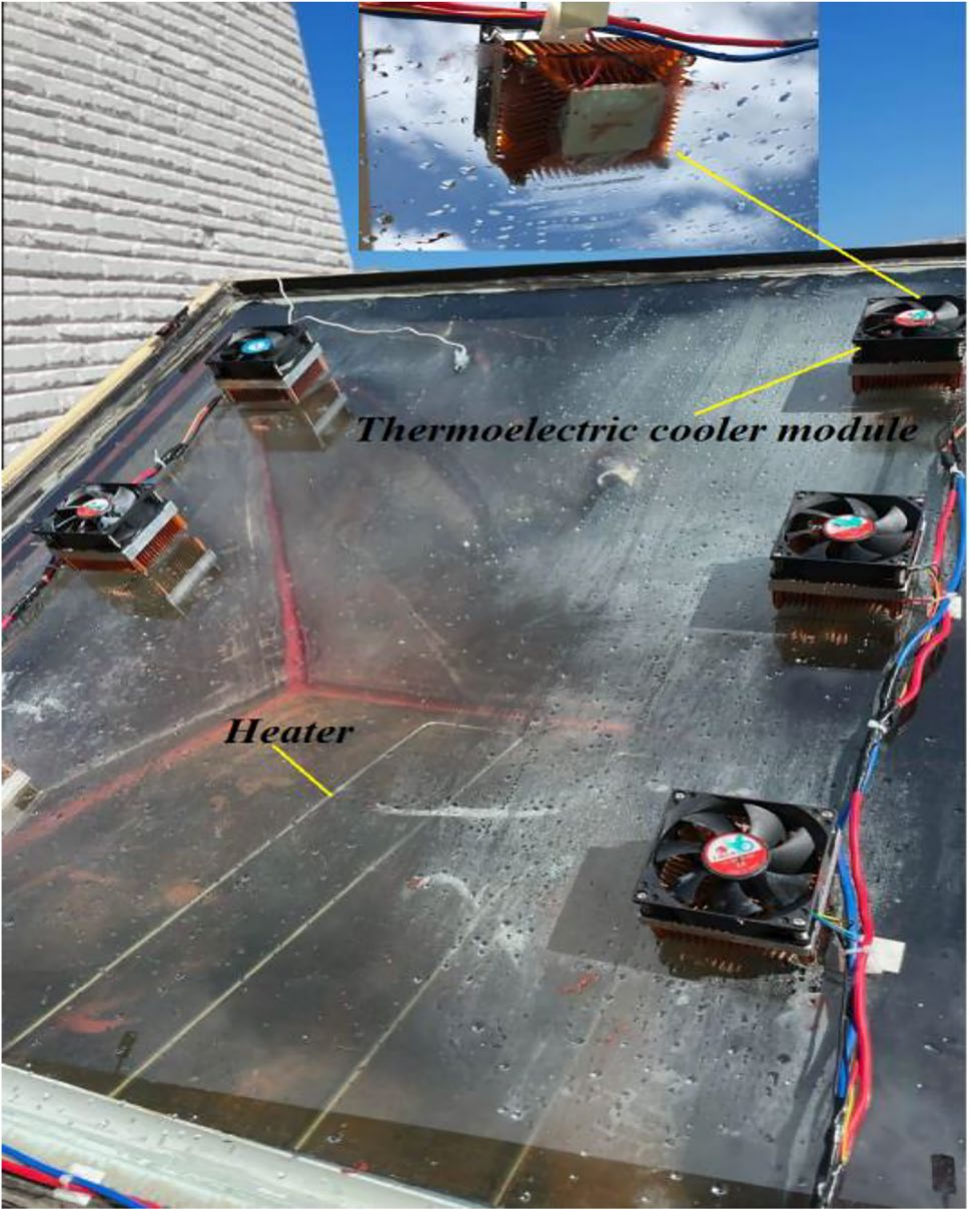
Photograph for the improved solar still with glass cooling and electric heater

Figure 3 shows six thermoelectric cooling modules (TEC1-12,706) that were installed on the glass in two columns to achieve a cooling effect. The operating voltage was set to 12 V and the operating current to 2A. Six thermoelectric chips measuring 40 mm long, 40 mm wide, and 3.4 mm high were fabricated and attached to the glass cover. The cold sides of the TECs served as coolers to remove heat from the 4-mm-thick solar still glass, while the hot side attached to the heat sink integrated with a cooling fan. The TEC modules were fixed to the glass using white silicone glue. The power consumption for each module was 24 W.

**Fig. 4:**
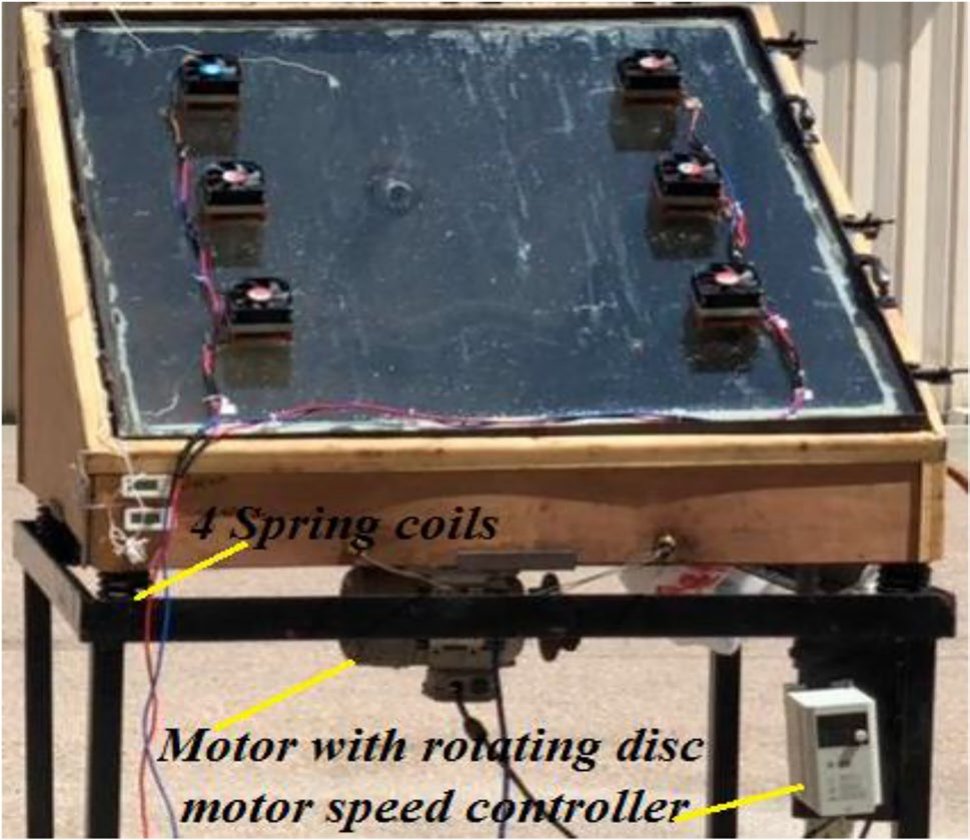
Schematic drawing for the vibration solar still with four coil springs

The vibration system was composed of a 1-hp power motor (maximum speed of 1380 rpm), a small disk with a 10-mm diameter mounted on the motor shaft, chassis, and four coil springs. Rotating an eccentric mass causes an unbalanced force, which leads to an oscillating force. Four coil springs were installed on the chassis to generate the degree of freedom of the system. The mechanical properties of the spring were as follows: 10 active coils, a free length of 150 mm, and a mean diameter of 55 mm (Fig. 4). A 0.6-kg mass was attached 50 mm eccentric from the center of the disk. The motor was centered at the bottom of the test rig with two fixed beams to generate the vibration of the system. An LS-type frequency inverter was used to change the motor speed, which was examined to establish the daily freshwater productivity.

**Fig. 5:**
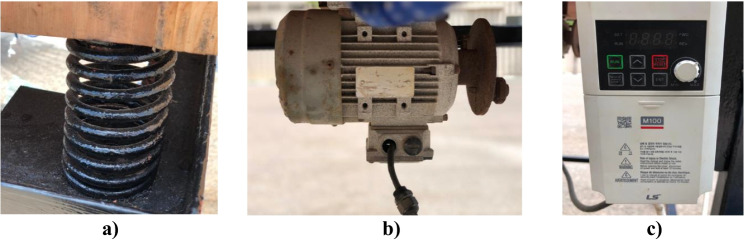
Photographs of the tools for vibration motion generator. **a**) spring; **b**) Motor with rotating disc; **c**) Motor inverter

Figure 5 shows the free body forces of the system. The balanced forces are as follows:

1 $$\sum \left[\mathrm{Inertia }+\mathrm{ external}\right]\mathrm{forces}=0$$

2 $$\left(M-m\right)x+\left(mx-m{\omega }^{2}e sin\omega t\right)+cx+kx=0$$

**Fig. 6:**
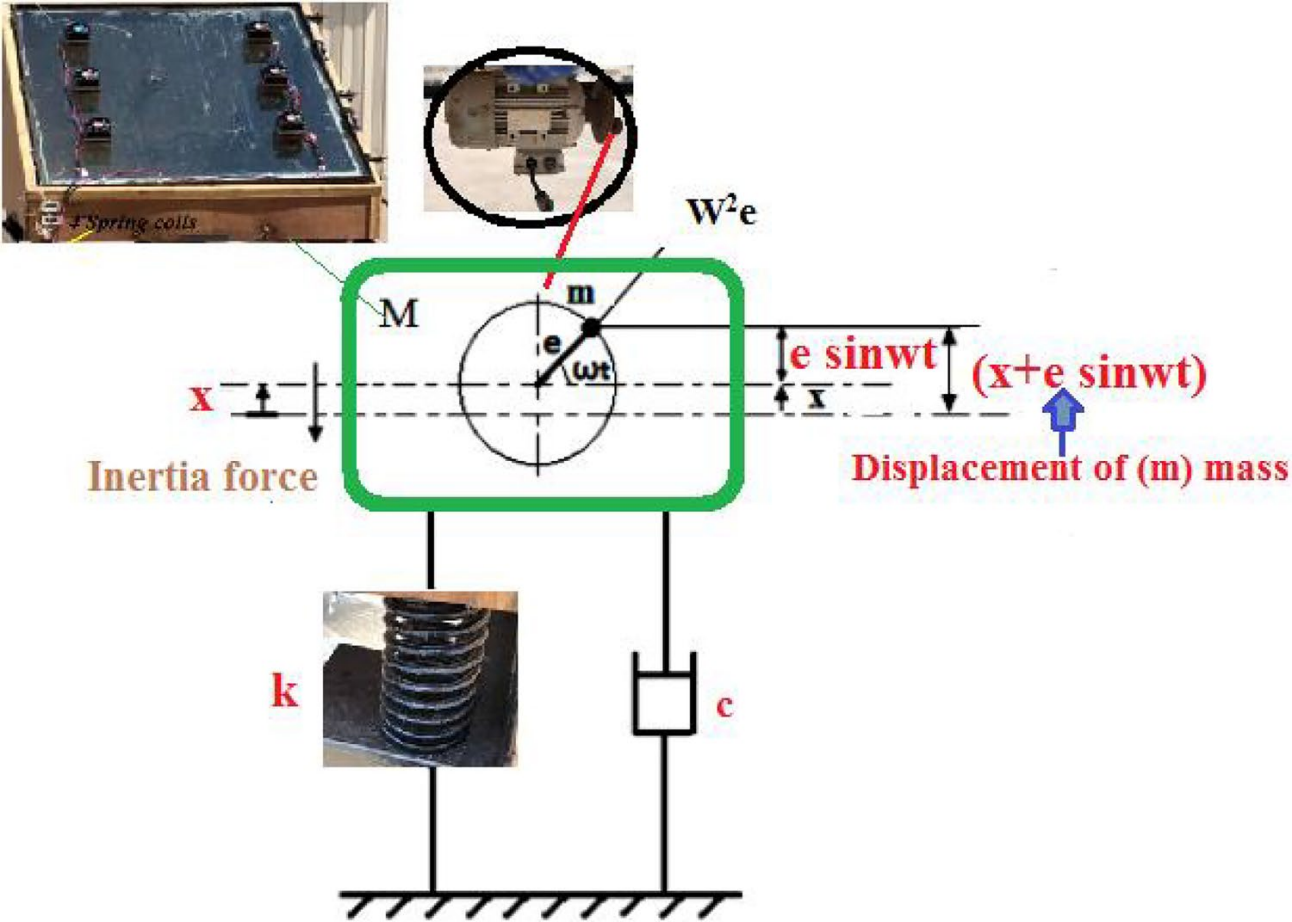
Displacement and forces due to unbalance mass rotating

Equation (2) shows the motion for the forced damped vibration under unbalanced rotation (Fig. 6).

## Measurement and error examination

**Fig. 7:**
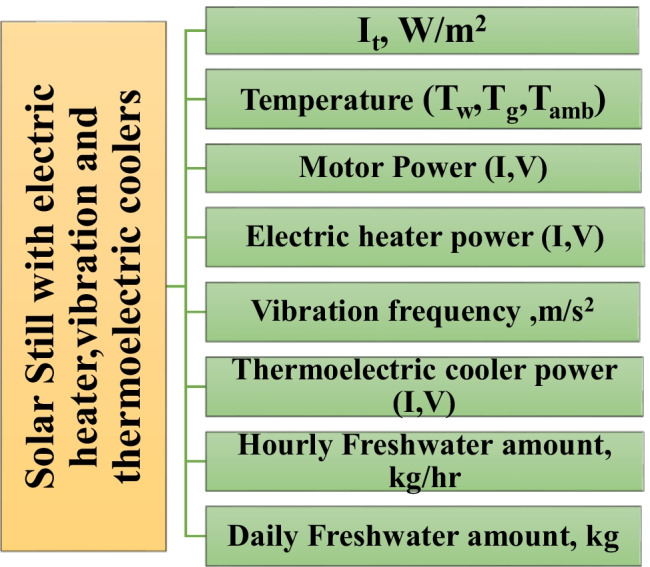
Photograph for the improved solar still with four coil springs

Figure 7 illustrates the steps of the measurement in the case of integrating the electric heater, vibration method, and TECs with the solar still. The common measurement steps in the five cases studied were as follows: solar radiation intensity (It), temperatures (ambient (Tamb)), glass cover (Tg), saline water (Tw), and hourly and daily purified water volume rates. The measurements were taken every 1 h. Four K-type thermocouples assimilated with a G4LCUEA modular programmable logic control were used for the temperature measurements. The total solar radiation was measured using a solarimeter. The collected freshwater was measured using a calibrated flask. The power values of the electric heater, vibration, and TECs were calculated by measuring the current and voltage. The acceleration was measured using a vibration meter (VM-6310). The motor speed was measured using a digital tachometer (model KM 2241). The measured acceleration was 1.14 G s.

Uncertainties and errors evolved as a result of the test environment and measurement devices throughout the testing process. Hence, the estimated metrics, such as thermal efficiency and daily mass flow rate, were computed from the measurement temperature, hourly water output, and solar radiation, which were precisely calculated and analyzed.

Consider a series of measurements *Z*_1_, *Z*_2_,…… for determining *n* as an empirical variable. Using these measurements, the following equation can be used to generate any desired *R* value for the experiment:

3 $$R=R({Z}_{1},{Z}_{2},{Z}_{3},\dots \dots ,{Z}_{n})$$

The uncertainty and errors for the devices were evaluated using Holman’s approach (1994). The following equation was used to determine the uncertainty WR in the obtained result:

4 $${W}_{R}={\left[{\left(\frac{\partial R}{\partial {Z}_{1}}{W}_{1}\right)}^{2}+{\left(\frac{\partial R}{\partial {Z}_{2}}{W}_{2}\right)}^{2}+\dots \dots \dots \dots \dots \dots \dots \dots + {\left(\frac{\partial R}{\partial {Z}_{n}}{W}_{n}\right)}^{2}\right]}^\frac{1}{2}$$

where *W*_1_, *W*_2_, *W*_3_,….….*W*_n_ represent the independent variables of the uncertainties. The calculated parameters showed uncertainty values of ± 1.47% for the daily freshwater output. Table 1 provides a list of all the measured values for the tested parameters.

**Table 1:** Errors for various experimental devices

Device	Range	Accuracy	%error
Thermocouples type (K)	0–100 °C	± 0.1 °C	1.7
Solarimeter	0–2000 W/m^2^	± 1 W/m^2^	0.15
Calibrated tank	0–2500 mL	± 5 mL	0.89
Ammeter	0–20 A	± 0.1 A	0.1
Voltmeter	0–100 V	± 0.1 V	0.23
Model KM 2241 digital tachometer	1–1000 RPM	0.1 RPM (0.5–1000) RPM	± (0.05% + 1 digit)
Vibration meter type VM-6310	0.1 ~ 400 m/s^2^	< 5%	± 1.4%

## Results and discussions

Practical results were obtained by studying the individual influences of the electric heater, vibration motion, and TEC; the effect of two of these parameters; and the effect of all three parameters during the summertime.

The vibration motion was examined at different frequencies as the motor speed was changeable. The optimum motor speed resulted in the highest freshwater productivity and prevented the water in the basin from splashing onto the glass cover. The experimental study was conducted over a period of several days in each month during the summer and winter seasons. The experiments were performed at water depths of 10 mm.

### Effect of using an electric heater

**Fig. 8:**
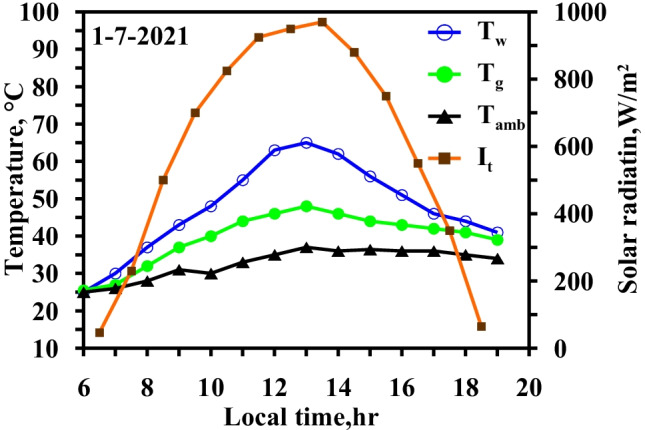
Hourly solar radiation and temperature variation for conventional solar still at summer monthsc

Figure 8 demonstrates the variation in the water and glass temperatures in the CSS. During summer, the maximum solar intensity is at 13:00 local time and reaches 970 W/m^2^, and the sunlight period exceeds 12 h. The water basin and glass temperatures vary from 25 to 86 °C and from 25 to 58 °C, respectively. The temperature differential between the water basin and the glass reaches a maximum value of 32 °C at 18:00 local time. An electric heater is powered by solar energy through solar cells, and the solar energy is turned into electricity through the solar cells and then stored in batteries for utilization after sunset.

The electric heater ran for 9 h, keeping the water basin temperature between 80 °C and 84 °C for 8 h.

**Fig. 9:**
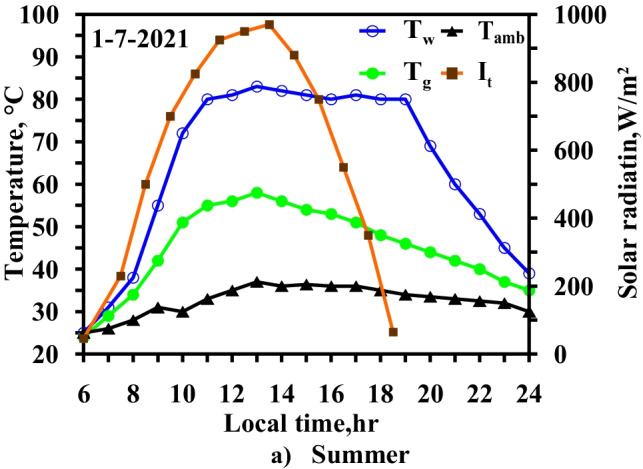
Hourly solar radiation and temperature variation for modified solar still with heater at summer months

Note that the results shown in Figs. 8 and 9 were measured on the same days to assess the effectiveness of using the heater technique on freshwater productivity.

Figure 9 shows that the use of the electric heater led to an increase in the water temperature and vapor pressure that caused the increase in the evaporation rate. Moreover, the increase in the water temperature relative to the conventional system reached 48.3% and 88% at noon and during the day, respectively.

#### Influence of usage of electric heater and thermoelectric coolers

**Fig. 10:**
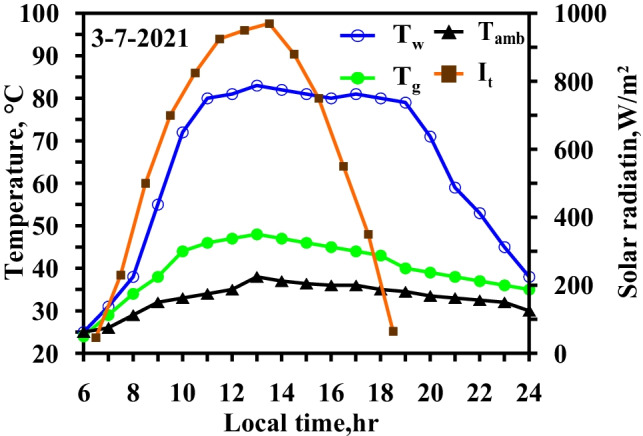
Hourly solar radiation and temperature variation for modified solar still with heater and thermoelectric coolers at winter and summer months

Figure 10 depicts the meteorological data for the weather conditions, including the solar radiation and ambient temperature glass, as well as the temperature fluctuation in the glass and water basin in local time, utilizing two different approaches, namely, the use of TECs for glass cover cooling and the use of an electric heater in summertime.

The results indicated the effect of using TECs on the glass temperature in the summertime. The comparison of the results in Figs. 9 and 10 clearly revealed that the use of the TECs exerted a significant effect on the glass temperature. The use of the electric heater in the still increased the water basin temperature, but the freshwater productivity still depended on the temperature difference between the water basin and the glass; this difference led to the cooling of the glass cover. The maximum temperature difference reached 37 °C at 18:00 local time, and the temperature difference fell in the range of 32–37 °C during the operation of the heater. As the glass temperature decreased, the condensation rate increased.

#### Effect of usage of electric heater and vibration motion

**Fig. 11:**
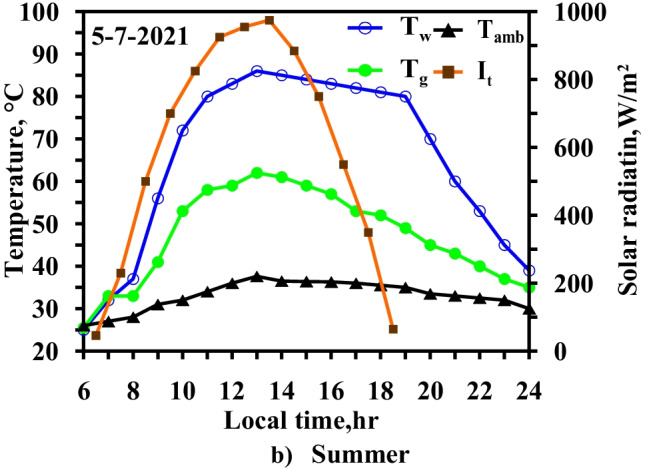
Hourly solar radiation and temperature variation for modified solar still with heater and vibration at summer months

The variations in ambient temperature, solar radiation, water temperature, and glass temperature when the electrical heater and vibration motion were integrated are shown in Fig. 11.

After studying the effect of using the electric heater, the heat transfer rate had to be increased, especially with the increase in water basin temperature. The vibration motion technique was combined with the electric heater to increase the evaporation rates for the water basin’s surface, where the heat transfer mechanism shifted from natural convection to forced convection.

Figure 10 clearly shows that the temperature difference between the water basin and the glass surface varied from 22 to 29 °C in the case of the integrated electric heater and vibration motion. As for the case involving the use of the electric heater only, the temperature difference was within 25 °C–32 °C during the heater’s operation. The comparison was performed from 11:00 to 18:00 local time as the water basin temperature was approximately in the range of 80–85 °C. The temperature difference decreased as the glass temperature increased as a result of the evaporation rate increasing due to the vibration motion. The glass temperature was increased more than in the case of an integrated heater alone which indicates that the evaporation rate increases. The vibration motion increased the heat transfer rate as the forced convection was subjugated.

The vibration effect clearly showed a significant effect on the increase in the evaporation rate. Meanwhile, the condensation rate decreased as the glass temperature increased.

**Fig. 12:**
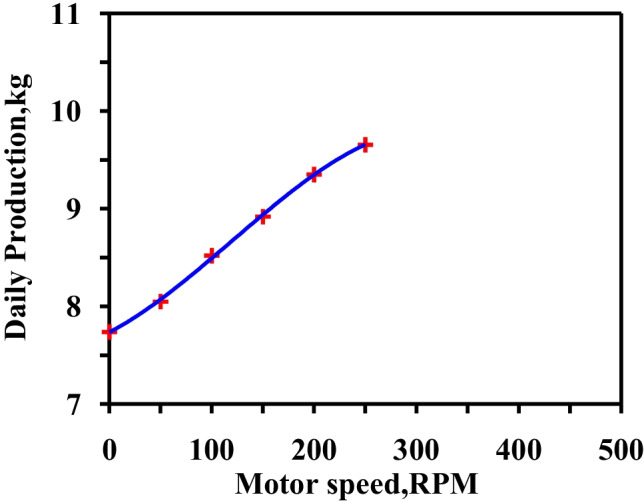
Hourly solar radiation and temperature variation for modified solar still with heater and vibration at summer months

Figure 12 depicts the effect of motor speed on daily productivity. The maximum speed is 250 RPM, as beyond that value, the water splashed on the glass.

#### Effect of usage of an electric heater, vibration motion, and thermoelectric coolers

**Fig. 13:**
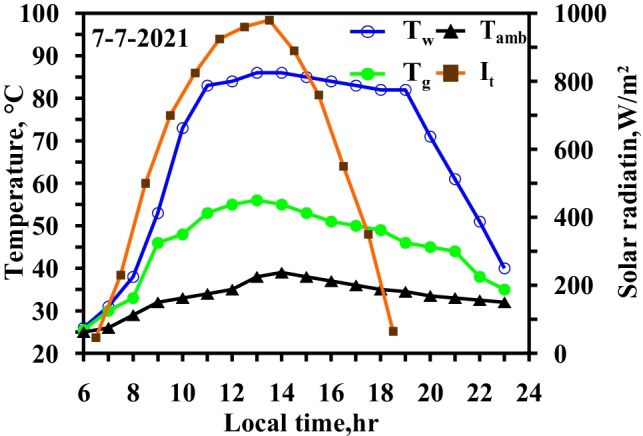
Hourly solar radiation and temperature variation for modified solar still with heater and thermoelectric coolers and vibration at summer months

Figure 13 depicts the climatic conditions, including solar radiation, ambient temperature, and temperature variation, for the water and glass of the modified solar still that utilizes the heater, vibration motion, and TECs during local time in the winter and summer periods.

The temperature difference between the water basin and the glass surface varied from 29 to 33 °C. In the case of the integrated electric heater and TECs, the temperature difference was within 32–37 °C.

Nevertheless, boosting the evaporation rate was insufficient to enhance freshwater productivity because high evaporation rates without suitable condensation rates produce a large amount of vapor within the solar still. The vapor offers no benefit, thus rendering the first two strategies useless. Therefore, the integration of the TECs was an important strategy for the success of the two previous methods.

## Hourly and daily freshwater productivity

**Fig. 14:**
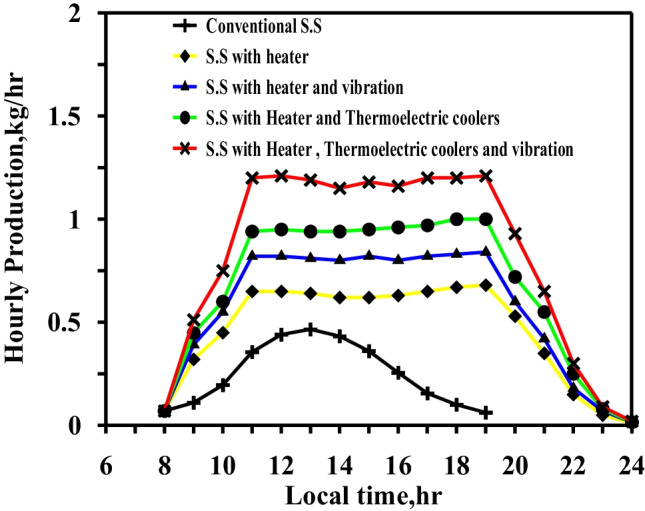
Hourly production of freshwater for different cases summer times

Figure 14 shows that the CSS had a maximum productivity value of 0.466 kg/h at 13:00 local time and that the productivity duration approached 11 h depending on the solar intensity during the day. Comparing the productivity in the five scenarios with the conventional productivity of the still at midday revealed that the rates of increase with the integration of the electric heater, heater with vibration, heater with TECs, and heater with vibration and TECs were 47.4%, 70.1%, 104%, and 134.3%, respectively. The usage of the heater for long periods caused the water temperature to increase to 80–85 °C for 8 h, resulting in high hourly production, especially between 11:00 and 19:00.

**Fig. 15:**
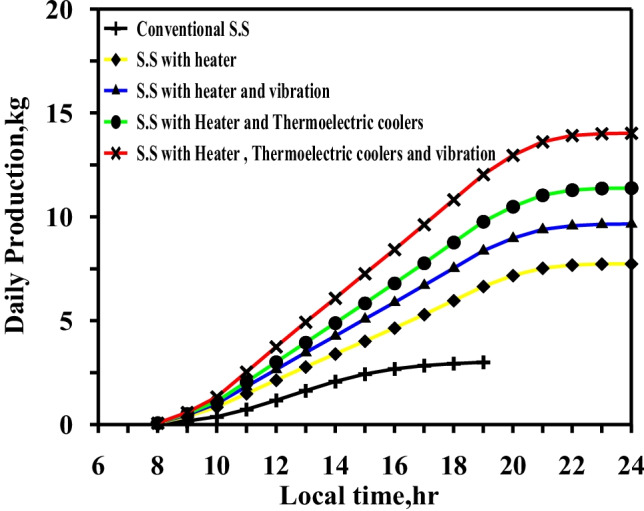
Comparison of accumulated production of the freshwater for different cases at summer times

Figure 15 displays a comparison between the daily total results for the five different cases. The productivity of the four cases increased by 157%, 208%, 262%, and 327% relative to the conventional still.

The boost in daily desalinated water was expected as increasing the water temperature to 80–85 °C for 8 h led to a significant increase in evaporation rates. The integration of vibratory movement assisted in enhancing the rate of evaporation by transferring heat from a natural to a forced mechanism, thus maximizing evaporation rates. The glass cooling method was designed to increase the condensation rate of the glass to nearly the rate of evaporation in order to achieve the greatest effect from the two techniques.

**Fig. 16:**
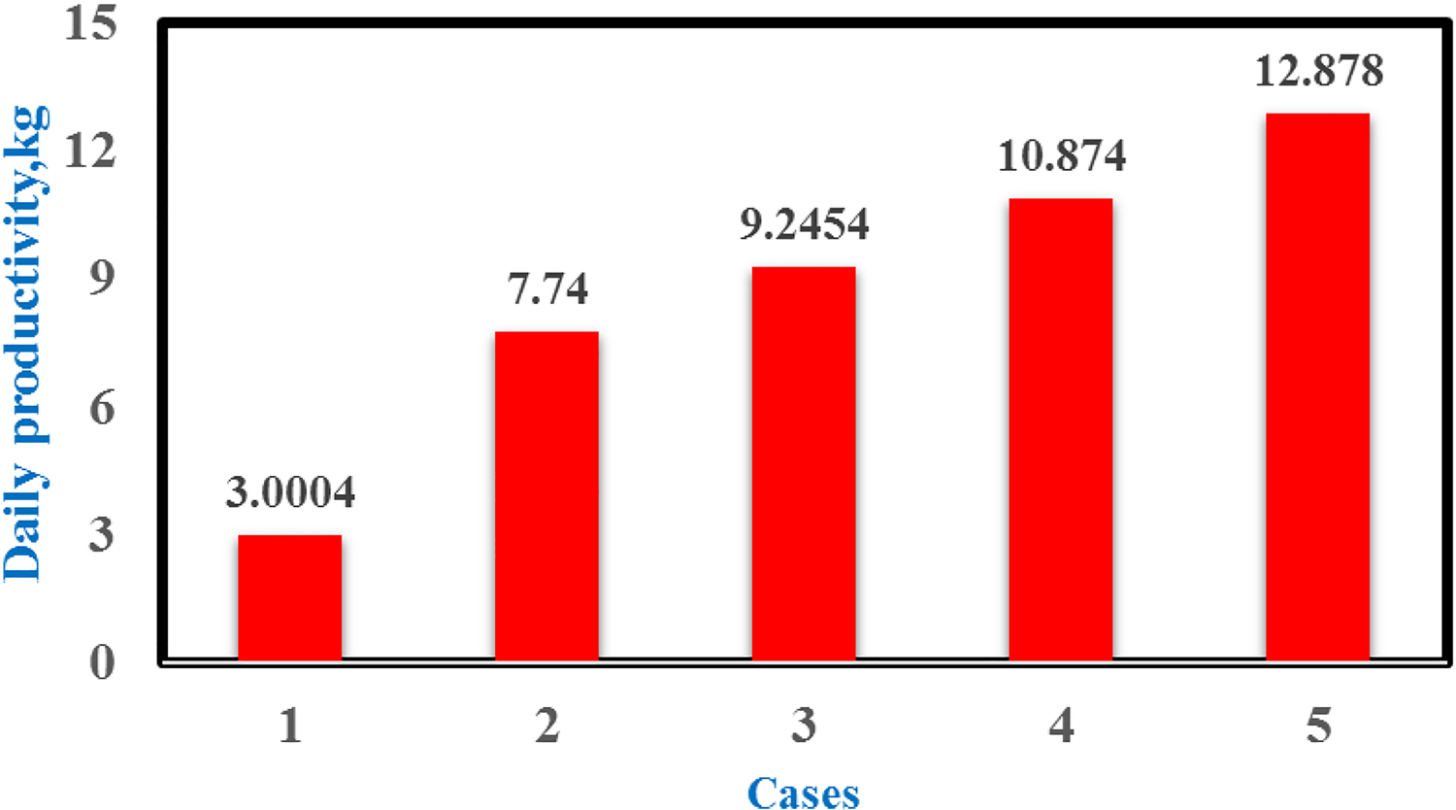
Comparison of daily accumulated production of the fresh water for five different cases

Figure 16 summarizes the effect on the daily freshwater productivity of the different modifications using the electric heater, vibration motion, and glass cover cooling.

The following equations show the system efficiency of the modified solar still:

5 $${\eta }_{\mathrm{system}}=\frac{\sum {m}_{p} \times {h}_{fg}}{\left(A \times I\left(t\right) \times 3600\right)+We }$$

**Fig. 17:**
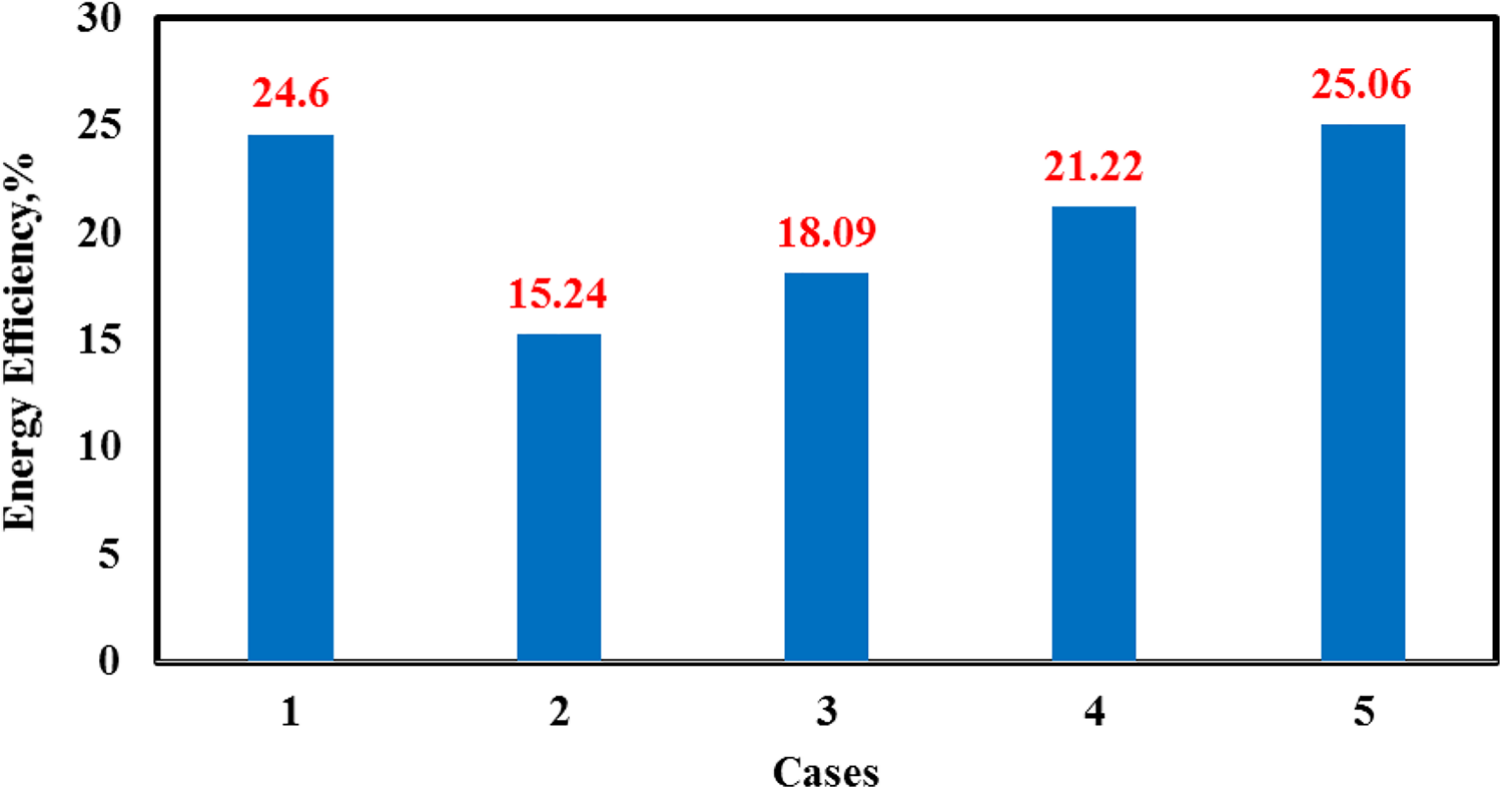
Comparison of daily efficiency of the fresh water for five different cases

Figure 17 summarizes the effect on daily efficiency of the different modifications using the electric heater, vibration motion, and glass cover cooling. The daily efficiency for the first three modified cases decreased relative to the conventional case as the area subjected to solar energy increased with the introduction of two PV solar cells. The hourly freshwater production increased in case (5) relative to the conventional case.

### Exergy analysis

The exergy balance is analyzed using the thermodynamics law. The exergy analysis is the relationship between the generated exergy and the sum of exergy input into solar desalination, which is determined using [38]:

6 $${\eta }_{ex}=\frac{{Ex}_{out}}{{Ex}_{in}}$$

The solar desalination exergy product is calculated as follows [26]:

7 $${Ex}_{in}=\left(A \times I\left(t\right) \times \left(1-\frac{4{T}_{a}}{3{T}_{S}}\right)+\frac{1}{3}{\left(\frac{{T}_{a}}{{T}_{s}}\right)}^{4}\right)+We$$

where *Ts* shows the temperature of the sun (equal to 5727 °C). The exergy product of the solar still could be calculated through:

8 $${Ex}_{out}=\left(\frac{{\dot{m}}_{ev}}{3600}\times {h}_{fg}\times \left(1-\frac{{T}_{a}}{{T}_{w}}\right)\right)$$

**Fig. 18:**
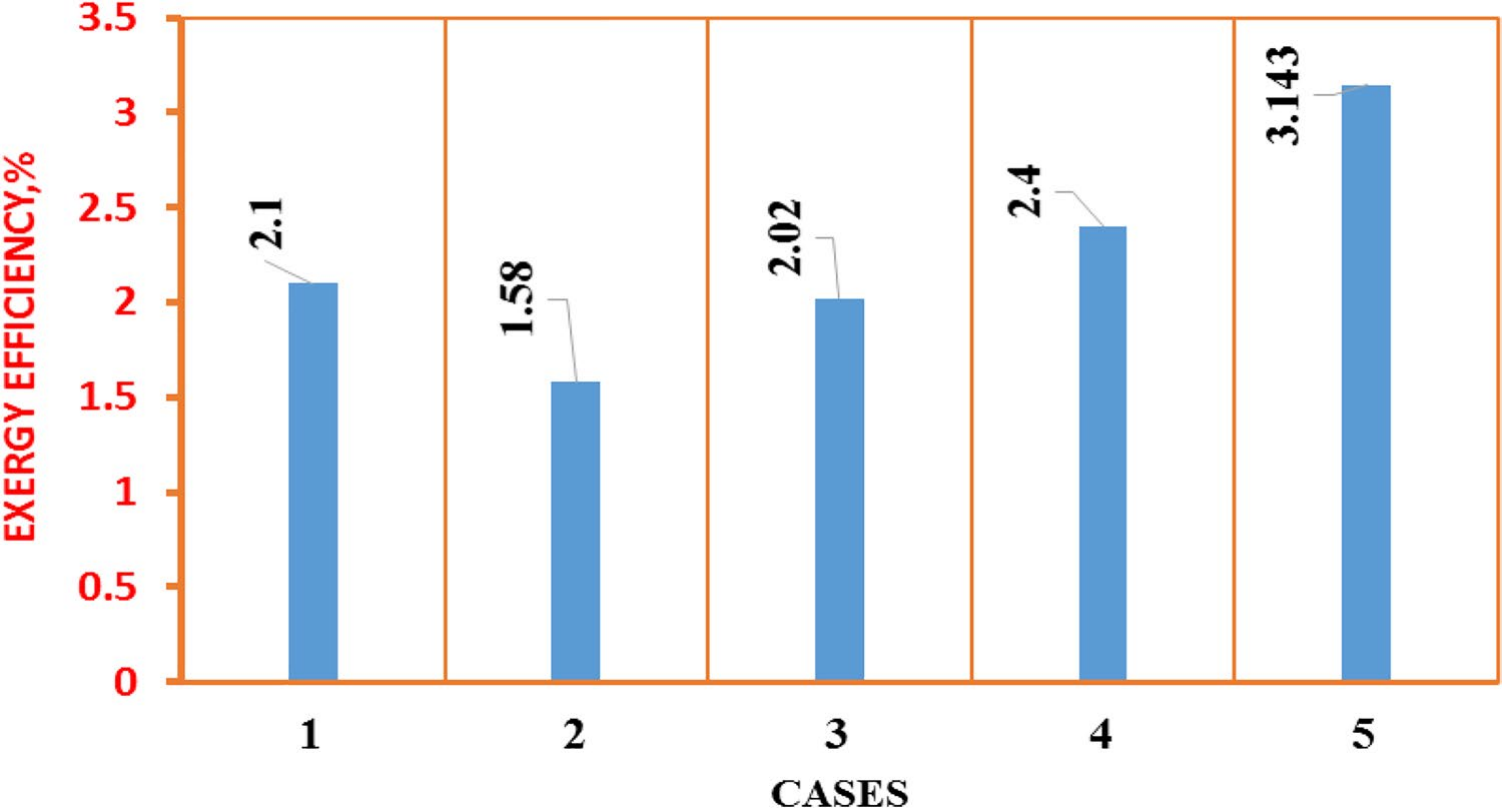
Comparison of exergy efficiency of the fresh water for five different cases

Figure 17 represents the average daily energy efficiency for the five solar still cases. As shown, the energy efficiency for the case (5) is the highest as the daily freshwater productivity is the highest. The results showed that the exergy efficiency in the case (5) was the highest also due to higher productivity as shown in Fig. 18.

## Cost analysis

In this section, financial viability is explored to determine the sustainability of the suggested method. Several techniques were used with consideration of the main factors affecting the cost of purified water, such as site, fed water quality, system capacity, and price of components (Chaichan and Kazem 2015; Praveen kumar et al. 2018; Rahbar et al. 2017; Rajaseenivasan et al. 2014) to measure the economy of the modified solar still (Al-Garni 2012a; Chaichan and Kazem 2015; Praveen kumar et al. 2018; Rajaseenivasan et al. 2014, 2016). The techniques were the capital recovery factor (CRF) and the annual/annual/fixed/cost (FAC) factor. The values were calculated as follows:

9 $$CRF =\frac{{\text{i(}{1} \, \text{+} \, \text{i)}}^{\text{N}}}{{\left({1} \, \text{+} \, {\text{i}}\right)}^{\text{N}} - \, {1}}$$

10 $$FAC = CS \times \left(CRF\right)$$

The salvage value of the entire apparatus after its lifetime (*N* = 10 years) was taken as equal to 20% of the total fixed cost. Moreover, the sinking fund factor (SFF) and the annual/yearly salvage value (ASV) were calculated as follows (G Manuel 2009):

11 $$SFF=\frac{i}{{\left(i + 1\right)}^{n}-1}$$

12 $$ASV = SFF \times S$$

Accordingly, the maintenance cost covered the cost of cleaning the basin and the cover from salts, along with the maintenance costs of the six thermoelectric modules and fans and the PV panel. The annual servicing cost (ASC) was assumed to be 15% of the fixed yearly cost.

13 $$ASC = 0.15 \times FAC$$

For the yearly cost, the estimation was

14 $$TAC = FAC + ASC - ASV$$

Finally, the cost per liter ($\mathrm{CPL}$) was calculated as follows:

15 $$CPL = \frac{TAC}{ L}$$

The number of cloudy and rainy days [Nc] in Alexandria will never surpass 15 days according to the Egyptian Solar Atlas. Thus, the operational days were considered to be 350 days per year in this study. Table 2 shows the pricing of the system components as they are marketed in the Egyptian market. Table 3 shows the cost of the traditional solar still. The estimated CPL for the traditional case was 0.0192 $/L/m^2^, whereas that for the modified scenario (5) was 0.01786 $/L/m^2^.

**Table 2:** Cost of components

	Conventional solar still		Proposed solar still	
Component	Quantity	Total cost ($)	Quantity	Total cost ($)
Solar still	1	75	1	75
Thermoelectric with fan	-	-	6	10
Vibration motor	-	-	1	25
PV panel	-	-	2	120
Inverter (1500 W, 220 V, 20A)	-	-	1	80
MPPT controller charger	-	-	1	60
Heater (450 W)	-	-	1	10
Control unit	-	-	1	5
Accessories	-	-	2	21
Σ Total		75		407

**Table 3:** Economic analysis results

Calculated parameters	Equation	Conventional solar still	Proposed solar still (case 5)
Life, *n*		10	10
Interest per year i,%		12	12
Capital cost (CS), ($)		75	407
Salvage value (S), ($)	*S* = *0.2* × *CS*	15	81.4
Capital recovery factor (CRF)	$$CRF= \frac{i {(1+i)}^{n}}{{(1+i)}^{n}-1}$$	0.177	0.177
Sinking fund factor, (SFF)	$$SFF=\frac{i}{{\left(i+1\right)}^{n}-1}$$	0.057	0.057
Fixed annual cost (FAC), ($)	$$FAC=CRF \times CS$$	13.275	72.039
Annual operating and maintenance cost AMC, ($)	*AMC* = *0.15* × *FAC*	1.99125	10.80585
Annual salvage value (ASV), ($)	$$ASV=SFF \times S$$	0.855	4.6398
Total annual cost TAC, ($)	*TAC* = *FAC* + *AMC –ASV*	14.41125	78.20505
Annual freshwater productivity, L		748	4378.52
Annual cost per 1 L of distilled water productivity, CPL, ($/L)	*CPL* = $$\frac{TAC}{ L}$$	0.019266377	0.017861069

Table 4 shows the CPL for the five cases at different years. Table 5 relates the current study’s results to those estimated in prior research (G Manuel, 2009; Morad et al. 2015; Rajaseenivasan et al. 2016; Yadav and Sudhakar, 2015). The results show a clear agreement with the literature.

**Table 4:** The CPL of solar desalinations with different lifetimes and interest rates

Solar still	*n*/years	*i* %	CRF	FAC	SSF	S	ASV	AMC	TAC	M	CPL
Case (1)	10	0.12	0.17	13.3	0.056	15	0.85	1.99	14.4	748	0.018
	10	0.2	0.23	17.88	0.038	15	0.57	2.68	19.9	748	0.026
	20	0.12	0.13	10.04	0.013	15	0.21	1.51	11.3	748	0.014
	20	0.2	0.21	15.4	0.005	15	0.08	2.31	17.6	748	0.023
Case (2)	10	0.12	0.17	65.83	0.056	74.4	4.23	9.87	71.5	2554	0.027
	10	0.2	0.23	88.7	0.038	74.4	2.86	13.3	99	2554	0.038
	20	0.12	0.13	49.8	0.013	74.4	1.03	7.47	56	2554	0.022
	20	0.2	0.21	76.3	0.005	74.4	0.39	11.45	87.3	2554	0.036
Case (3)	10	0.12	0.17	70.26	0.056	79.4	4.52	10.5	76.2	3049	0.025
	10	0.2	0.23	94.7	0.038	79.4	3.05	14.2	105	3049	0.034
	20	0.12	0.13	53.14	0.013	79.4	1.1	7.97	60	3049	0.019
	20	0.2	0.21	81.5	0.005	79.4	0.42	12.2	93.3	3049	0.03
Case (4)	10	0.12	0.17	67.6	0.056	76.4	4.35	10.1	73.4	3755	0.019
	10	0.2	0.23	91.1	0.038	76.4	2.94	13.66	108	3755	0.027
	20	0.12	0.13	51.14	0.013	76.4	1.06	7.67	57	3755	0.015
	20	0.2	0.21	78.44	0.005	76.4	0.4	11.76	58.5	3755	0.016
Case (5)	10	0.12	0.17	72.03	0.056	81.4	4.63	10.8	78.2	4620	0.017
	10	0.2	0.23	97	0.038	81.4	3.13	14.56	108	4620	0.023
	20	0.12	0.13	54.4	0.013	81.4	1.12	8.17	61.5	4620	0.013
	20	0.2	0.21	83.5	0.005	81.4	0.43	12.53	95.6	4620	0.021

**Table 5:** Comparison between present study and different solar still studies

Ref	Type of solar still	Max yearly yield (L/m^2^)	Cost per liter ($/L/m^2^)
Present study	Solar still with electric heater, thermoelectric, vibration motion	4378	0.017861069
Rahbar et al. 2017	Using thermoelectric	912	0.1422
Nazari et al. 2019	With thermoelectric and nanofluid	1689	0.0252
Suneesh et al. 2014	With cooling glass cover	1196	0.0421
Abdullah et al. 2021	Wick corrugated absorber, nano-enhanced phase change material, and photovoltaics-powered heaters	2108	0.025

### Benefit cost ratio

The benefit cost ratio method is one of the engineering economics strategies for comparing designs in terms of cost. The approach is a realistic and well-known method for appraising projects that involves economic analysis of private investment initiatives. The benefit cost ratio is attained via (Kosmadakis et al. 2009):

16 $$BCR=\frac{UAB}{TAC}$$

The current value of benefit in solar desalination can be gained through the following equation (Shoeibi et al. 2022c):

17 $$UAB= K\times POW$$

where *POW* stands for the price of water, which varies by country and has been 0.28 $ per liter in Egypt. For an investment to be efficient, the benefit-to-cost ratio must be greater than one.

### Exergoeconomic parameter

The exergoeconomic parameter is described as the optimization of the cost analysis while taking into account the system’s exergy analysis, and it is derived using the following formula (Shoeibi et al. 2022c):

18 $$\mathrm{R EN }= \frac{{\left({E}_{en}\right)}_{out}}{TAC}$$

19 $$\mathrm{R Ex}= \frac{{\left({E}_{ex}\right)}_{out}}{TAC}$$

### Environmental parameter

During this step, all parameters were evaluated based on the value of CO_2_ pollution reduction achieved by the solar still. However, most of the components of a solar sill, such as iron sheets, aluminum sheets, Plexiglas, and pipes, electricity generated from fossil fuels, which is damaging to the environment. During the manufacturing of these parts, a huge amount of highly dangerous and damaging chemicals are released into the environment (Srithar et al. 2016). CO_2_ removal and CO_2_ emission are used to determine environmental economic parameters.

#### ***CO***_***2***_*** emissions***

CO_2_ production and distribution per kWh are around 0.96 kg [46]. Furthermore, the CO_2_ output per kWh is equal to 2 kg due to transmission loss (20%) and distribution loss (40%) caused by improper equipment. The annual CO_2_ emissions and lifetime CO_2_ emissions of solar desalination are calculated as follows:

20 $$ACDE = \frac{2\times {E}_{in}}{N}$$

21 $$CDED = 2\times {E}_{in}$$

#### ***CO***_***2***_*** reduction***

The annual CO_2_ reduction by solar desalination (kg CO_2_ per year) is approximately *(Een)*_*out*_ × 2. As a result, the CO_2_ reduction during a lifetime might be expressed as *(Een)*_*out*_ ×2× *n*. The following equation (Shoeibi et al. 2022c) is used to calculate the net amount of CO_2_ reduction per ton throughout a lifetime:

22 $$EP=\frac{2\left({\left({E}_{en}\right)}_{out}\times N-{E}_{in}\right)}{1000}$$

### Enviroeconomic parameter

The enviroeconomic parameter is defined as the cost of reducing CO_2_ during the lifespan of solar desalination. The cost of CO2 per ton was calculated at approximately 14.5$ per ton (Shoeibi et al. 2022c):

23 $$EPP=\frac{2\left({\left({E}_{en}\right)}_{out}\times N-{E}_{in}\right)}{1000}\times \mathrm{cost\;of\;CO}2\mathrm{\;per\;ton}$$

### Exergoenvironmental analysis

The exergoenvironmental parameter analyzes carbon dioxide emission reduction based on exergy output in single-slope solar desalination and is calculated as follows (Shoeibi et al. 2022c):

24 $$EPx=\frac{2\left({\left({E}_{ex}\right)}_{out}\times N-{E}_{in}\right)}{1000}$$

### Exergoenviroeconomic parameter

Exergoenviroeconomic analysis is a technique for calculating the cost of CO_2_ reduction while accounting for exergy.

25 $$EP{P}_{X} =\frac{2\left({\left({E}_{en}\right)}_{out}\times N-{E}_{in}\right)}{1000}\times \mathrm{cost\;of\;CO}2\mathrm{\;per\;ton}$$

Table 6 shows the benefit cost ratio for the five cases with different interest rates and lifetimes. The benefit cost ratio in the five cases was higher than unity. The results inferred that the benefit cost ratio in the case (5) was approximately 13.7% higher than that in the conventional ones.

**Table 6:** Benefit cost ratio of solar stills

Solar still	*n*/years	*i* %	TAC	POW/$	*K*	UAB/$	B/C
Case (1)	10	0.12	14.4	0.28	748	209.44	14.54
	10	0.2	19.9	0.28	748	209.44	10.52
	20	0.12	11.3	0.28	748	209.44	18.53
	10	0.2	17.6	0.28	748	209.44	11.90
Case (2)	10	0.12	71.5	0.28	2554	715.12	10.00
	10	0.2	99	0.28	2554	715.12	7.22
	20	0.12	56	0.28	2554	715.12	12.77
	20	0.2	87.3	0.28	2554	715.12	8.19
Case (3)	10	0.12	76.2	0.28	3049	853.72	11.20
	10	0.2	105	0.28	3049	853.72	8.13
	20	0.12	60	0.28	3049	853.72	14.23
	20	0.2	93.3	0.28	3049	853.72	9.15
Case (4)	10	0.12	73.4	0.28	3755	1051.4	14.32
	10	0.2	108	0.28	3755	1051.4	9.74
	20	0.12	57	0.28	3755	1051.4	18.45
	20	0.2	58.5	0.28	3755	1051.4	17.97
Case (5)	10	0.12	78.2	0.28	4620	1293.6	16.54
	10	0.2	108	0.28	4620	1293.6	11.98
	20	0.12	61.5	0.28	4620	1293.6	21.03
	20	0.2	95.6	0.28	4620	1293.6	13.53

The embodied energy of the five cases is shown in Table 7. The conventional and case (5) required roughly 219.58 kWh and 3305 kWh of energy, respectively, to create different components. The case (5) solar still had 14 times more embodied energy than the traditional one, according to the findings.

**Table 7:** Embodied energy of various components of solar desalinations (Shoeibi et al. 2022c)

Type of solar still	Name of component	Energy density		Mass of component/kg	Embodied energy/kWh
		MJ kg^−1^	kWh kg^−1^		
Case (1)	Glass	31.5	28.3	4	113.2
	Body	25	6.9	10	69
	Insulation	55.6	15.44	0.5	7.72
	Basin coating	90	25	0.5	12.5
	PVC pipe	77.2	21.4	0.2	4.28
	Support (galvanized)	50	13.9	3	41.7
	Rubber gasket	11.83	3.28	0.6	1.968
Total embodied energy (kWh)					219.58
Case (2)	Glass	31.5	28.3	4	113.2
	Body	25	6.9	10	69
	Insulation	55.6	15.44	0.5	7.72
	Basin coating	90	25	0.5	12.5
	PVC pipe	77.2	21.4	0.2	4.28
	Support (galvanized)	50	13.9	3	41.7
	Photovoltaic panel	98,800		3 m^2^	2940
	Copper heater	100	27.7	2	55.4
Total embodied energy (kWh)					3212.512
Case (3)	Glass	31.5	28.3	4	113.2
	Body	25	6.9	10	69
	Insulation	55.6	15.44	0.5	7.72
	Basin coating	90	25	0.5	12.5
	PVC pipe	77.2	21.4	0.2	4.28
	Support (galvanized)	50	13.9	3	41.7
	Copper heater	100	27.7	2	55.4
	Photovoltaic panel	98,800		3 m^2^	2940
	motor	77.2	21.4	4	85.6
Total embodied energy (kWh)					3298
Case (4)	Glass	31.5	28.3	4	113.2
	Body	25	6.9	10	69
	Insulation	55.6	15.44	0.5	7.72
	Basin coating	90	25	0.5	12.5
	PVC pipe	77.2	21.4	0.2	4.28
	Support (galvanized)	50	13.9	3	41.7
	Copper heater	100	27.7	2	55.4
	Photovoltaic panel	98,800		3 m^2^	2940
	fan	77	21.4	0.1	7.7
Total embodied energy (kWh)					3220
Case (5)	Glass	31.5	28.3	4	113.2
	Body	25	6.9	10	69
	Insulation	55.6	15.44	0.5	7.72
	Basin coating	90	25	0.5	12.5
	PVC pipe	77.2	21.4	0.2	4.28
	Support (galvanized)	50	13.9	3	41.7
	Copper heater	100	27.7	2	55.4
	Photovoltaic panel	98,800		3 m^2^	2940
	Motor	77.2	21.4	4	85.6
	Fan	77	21.4	0.1	7.7
Total embodied energy (kWh)					3305

Table 8 depicts the exergoeconomic parameter for various lifetimes and interest rates, accounting for exergy and energy desalination. Because of the high annual energy and exergy output and low productivity in the case (2) solar still with heater, the exergoeconomic parameter in case (2) is the lower in different states. Furthermore, when comparing the case (5) to the conventional one, the exergoeconomic increases about 6.6% and 66%, respectively, in terms of energy and exergy.

**Table 8:** Exergoeconomic parameter for solar stills

Solar still	*n*/year	*i*	TAC	Annual *(E*_*en*_*)*_*out*_/kWh	Annual *(E*_*ex*_*)*_*out*_/kWh	*R*_*En*_/kWh ^$−1^	*R*_*Ex*_/kWh $^−1^
Case (1)	10	0.12	14.4	718	46.6	49.86	3.24
	10	0.2	19.9	718	46.6	36.08	2.34
	20	0.12	11.3	718	46.6	63.54	4.12
	20	0.2	17.6	718	46.6	40.80	2.65
Case (2)	10	0.12	71.5	2326	225	32.53	3.15
	10	0.2	99	2326	225	23.49	2.27
	20	0.12	56	2326	225	41.54	4.02
	20	0.2	87.3	2326	225	26.64	2.58
Case (3)	10	0.12	76.2	2361	287	30.98	3.77
	10	0.2	105	2361	287	22.49	2.73
	20	0.12	60	2361	287	39.35	4.78
	20	0.2	93.3	2361	287	25.31	3.08
Case (4)	10	0.12	73.4	2783	341	37.92	4.65
	10	0.2	108	2783	341	25.77	3.16
	20	0.12	57	2783	341	48.82	5.98
	20	0.2	58.5	2783	341	47.57	5.83
Case (5)	10	0.12	78.2	4160	421	53.20	5.38
	10	0.2	108	4160	421	38.52	3.90
	20	0.12	61.5	4160	421	67.64	6.85
	20	0.2	95.6	4160	421	43.51	4.40

Table 9 displays the economic and environmental metrics for the five cases solar stills throughout a 20-year lifetime. As can be observed, case (5) solar still had a lower exergoenvironmental parameter than case (1). During the life of the system, CO_2_ emissions were also found to be dependent on the energy and exergy output. According to the environmental analysis, case (5) and traditional solar stills reduced carbon dioxide emissions by 166.4 and 28.72 tons, respectively. Furthermore, the case (5) and case (1) had enviroeconomic parameters of 2316.69 $ and 410.14 $, respectively. The exergoenvironmental parameter of the case (5) and conventional solar stills was 148.3 $ and 20.73 $, respectively.

**Table 9:** Environmental and enviroeconomic parameter of solar stills

	Case (1)	Case (2)	Case (3)	Case (4)	Case (5)
Lifetime (years)	20	20	20	20	20
Embodied energy (kWh)	217.112	3212.5	3298	3220.2	3305
Annual energy output (kWh)	718	2326	2361	2783	4160
Annual exergy output (kWh)	46.6	225	287	341	421
CO_2_ emission during life time (kg)	434.224	6368	6368	6440.4	6610
CO_2_ reduction during lifetime (ton)	28.72	46.52	46.52	46.52	166.4
Environmental parameter (ton Co_2_)	28.285776	86.615	87.844	104.8796	159.79
Enviroeconomic parameter ($)	410.142	1255.91	1273.74	1520.75	2316.96
Exergoenvironmental parameter (ton Co_2_)	1.429776	2.575	4.884	7.1996	10.23
Exergoenviroeconomic parameter ($)	20.731752	37.3375	70.818	104.3942	148.335

## Conclusion and remarks

To improve the yield of purified water in a single solar still, this study employed two techniques for CSS to raise the water basin temperature and another technique to reduce the glass temperature. The technique using an electric heater was used to the increase the water basin temperature to 80–85 °C, while vibration motion was used to increase the evaporation rate as the forced convection dominated. The vibration technique was applied using an eccentric rotating unbalanced mass. The speed motor was varied to ensure the optimum vibrating frequency range and was precisely determined and examined to prevent the water from splashing onto the glass surface and the water condensation from dropping from the glass into the basin. The electric heater, motor, and thermoelectric modules were powered by electrical energy from a PV solar panel. The five cases studied were as follows: CSS and solar still integrated with electric heater; electric heater and vibration motion; electric heater and thermoelectric modules; and electric heater, vibration motion, and TECs. The essential parameters, including solar radiation, temperatures (ambient, glass cover, and water basin temperatures), and the amount of freshwater were recorded every hour. All experiments were successfully conducted in Alexandria, which is located in the northwest of Egypt, during summer from 06:00 to 24:00 local time. The data analysis results are summarized as follows:

- The rates of increase in daily productivity for the four cases relative to the CSS were 157%, 208%, 262%, and 327%.
- The daily efficiency values for the five cases were 24.6%, 15.24%, 18%, 21.22%, and 25.06%.
- The modified case (5) was the best modification among all modifications as the daily productivity reached 12.8 kg/day, with the highest daily efficiency in terms of CPL being 0.01786 $/L/m^2^, which is lower than 0.019266 $/L/m^2^ of the traditional case.

## Data Availability

Not applicable.

## List of symbols and subscripts

A: Surface area (m^2^)
AMC: Annual maintenance cost
ASV: Annual salvage value
CPL: Cost per liter ($/L)
CSS: Conventional solar still
CRF: Capital recovery factor
E: Energy (W)
Ein: Embodied energy (kWh)
Ex: Exergy (W)
EP: Environmental parameter (ton Co_2_)
EPX: Exergoenvironmental parameter (ton Co_2_)
EPP: Enviroeconomic parameter ($)
EPPX: Exergoenviroeconomic parameter ($)
FAC: Fixed annual cost
hfg: Latent heat (kJ/kg)
i: Interest rate (%)
K: Average annual productivity (L)
M: Total mass of solar still, including water (kg)
m: Unbalanced mass (g)
ṁ: Water production rate (L s^−1^)
n: Life time of the still (years)
Nc: Number of cloudy days
POW: Price of water ($)
S: Salvage value ($)
SFF: Sinking fund factor
TECs: Thermoelectric coolers
Ta: Ambient temperature ({K})
Ts: Solar temperature ({K})
Tw: Water temperature ({K})
UAB: Present worth of benefit ($)
$$\overline{\mathbf{X}}$$: Displacement of mass (M-m), mm
ϕ: Nanofluid volume fraction
